# Net Water Uptake Calculated in Standardized and Blindly Outlined Regions of the Middle Cerebral Artery Territory Predicts the Development of Malignant Edema in Patients With Acute Large Hemispheric Infarction

**DOI:** 10.3389/fneur.2021.645590

**Published:** 2021-03-12

**Authors:** Hai-Bin Xu, Yu-Fei Sun, Na Luo, Jia-Qi Wang, Guo-Can Chang, Lin Tao, Ben-Qiang Yang, Hui-Sheng Chen

**Affiliations:** ^1^Department of Neurology, General Hospital of Northern Theater Command, ShenYang, China; ^2^Department of Radiology, General Hospital of Northern Theater Command, ShenYang, China

**Keywords:** net water uptake, large hemispheric infarction, malignant edema, area under curve, prediction power

## Abstract

**Background and purpose:** Previous studies have demonstrated that Net Water Uptake (NWU) is associated with the development of malignant edema (ME). The current study aimed to investigate whether NWU calculated in standardized and blindly outlined regions of the middle cerebral artery can predict the development of ME.

**Methods:** We retrospectively included 119 patients suffering from large hemispheric infarction within onset of 24 h. The region of the middle cerebral artery territory was blindly outlined in a standard manner to calculate NWU. Patients were divided into two groups according to the occurrence of ME, which is defined as space-occupying infarct requiring decompressive craniotomy or death due to cerebral hernia in 7 days from onset. The clinical characteristics were analyzed, and the receiver operating characteristic curve (ROC curve) was used to assess the predictive ability of NWU and other factors for ME.

**Results:** Multivariable analysis showed that NWU was an independent predictor of ME (OR 1.168, 95% CI 1.041–1.310). According to the ROC curve, NWU≥8.127% identified ME with good predictive power (AUC 0.734, sensitivity 0.656, specificity 0.862).

**Conclusions:** NWU calculated in standardized and blindly outlined regions of the middle cerebral artery territory is also a good predictor for the development of ME in patients with large hemispheric infarction.

## Introduction

Stroke has become a leading cause of mortality and disability worldwide, and it brings huge economic costs and family burdens (1). Acute ischemic stroke accounts for about 80% of all types of stroke (2). Large hemispheric infarction (LHI) is defined as affecting the majority of or complete middle cerebral artery (MCA) territory with or without anterior cerebral artery and posterior cerebral artery involvement (3). It is a disastrous subtype of acute ischemic stroke, which may lead to life-threatening swelling (4). Furthermore, LHI patients with malignant edema (ME) develop a mortality rate of nearly 40 ~ 80% under standard treatment, while mortality of those without ME is nearly 5 ~ 25% (3, 5, 6). It has been demonstrated by previous studies that timely decompressive craniotomy may reduce the mortality of LHI patients with ME (7, 8). Thus, early identification of LHI patients at risk for ME should be anticipated (3, 9).

There have been several studies exploring valid predictors of ME in LHI patients, such as the National Institutes of Health Stroke Scale (NIHSS), presence of hyperdense artery sign, a higher level of blood glucose, decreased level of consciousness, early infarct signs, intracranial cerebrospinal fluid volume, fluid balance variations, collateral circulation (10–17).

Interestingly, in 2018, Broocks' team found that Net Water Uptake (NWU) on baseline Computed Tomography (CT) was an important predictor of ME in LHI patients (9). Since then, accumulating evidence demonstrates that NWU can be used as an important qualified biomarker of edema in ischemic stroke. For example, NWU was used to estimate final infarction volumes (18), which serves as an indicator of “tissue clock” instead of the real “time clock” (19), and predicted the effect of recanalization (20) and early bleeding risk after endovascular treatment, especially with low ASPECTS (21). However, the measurement of NWU in previous studies mainly depends on CT perfusion (CTP) (9, 18–21). However, not all stroke centers have access to CTP in clinical practice. In this study, we aimed to investigate, whether NWU calculated in standardized and blindly outlined regions of the MCA territory is a reliable predictor of ME in patients with LHI.

## Methods

### Population

The medical records and images of consecutive patients with LHI at Northern Theater General Hospital between October 9, 2017, and July 13, 2020, were reviewed retrospectively. This retrospective study was approved by an institutional review board and informed consent was waived. Patients were screened based on the following inclusion criteria: (1) acute ischemic stroke involving the anterior circulation with non-enhanced CT (NECT) with or without computed tomography angiography (CTA) at admission within 24 h from symptom onset; (2) follow-up NECT or diffusion weighted imaging (DWI) available within 24–48 h from symptom onset; and (3) MCA infarction occupying >1/2 MCA territory confirmed by follow-up CT or DWI; (4) NIHSS >3 at admission. Patients were excluded based on the following exclusion criteria: (1) presence of intracranial or subarachnoid hemorrhage at admission; (2) presence of symptomatic intracranial hemorrhage (SICH) (22) in follow-up CT; (3) preexisting stroke with mRS ≥2; (4) images not qualified enough for measurement due to artifacts; and (5) patients undergoing mechanical thrombectomy (considering the effect on the development of ME). The enrolled patients were separated into the ME group and the non-ME group depending on the presence or absence of ME. According to a previous study (9), ME was defined as a space-occupying infarct requiring decompressive craniotomy or death resulting from cerebral hernia in 7 days from symptom onset.

The following characteristics of patients were recorded at baseline: gender, age, NIHSS, intravenous thrombolysis or not, previous medical history such as atrial fibrillation, hypertension, diabetes, and ischemic stroke, systolic and diastolic blood pressure, blood glucose, the time from onset to the first image.

### Image Acquisition and Analysis

CT scanner (General Electric, Boston, United States of America) was used in this study. The tube settings for NECT were 120 kV and 300 mA per rotation. Slices were reconstructed with a thickness of 5 mm.

**Figure 1:**
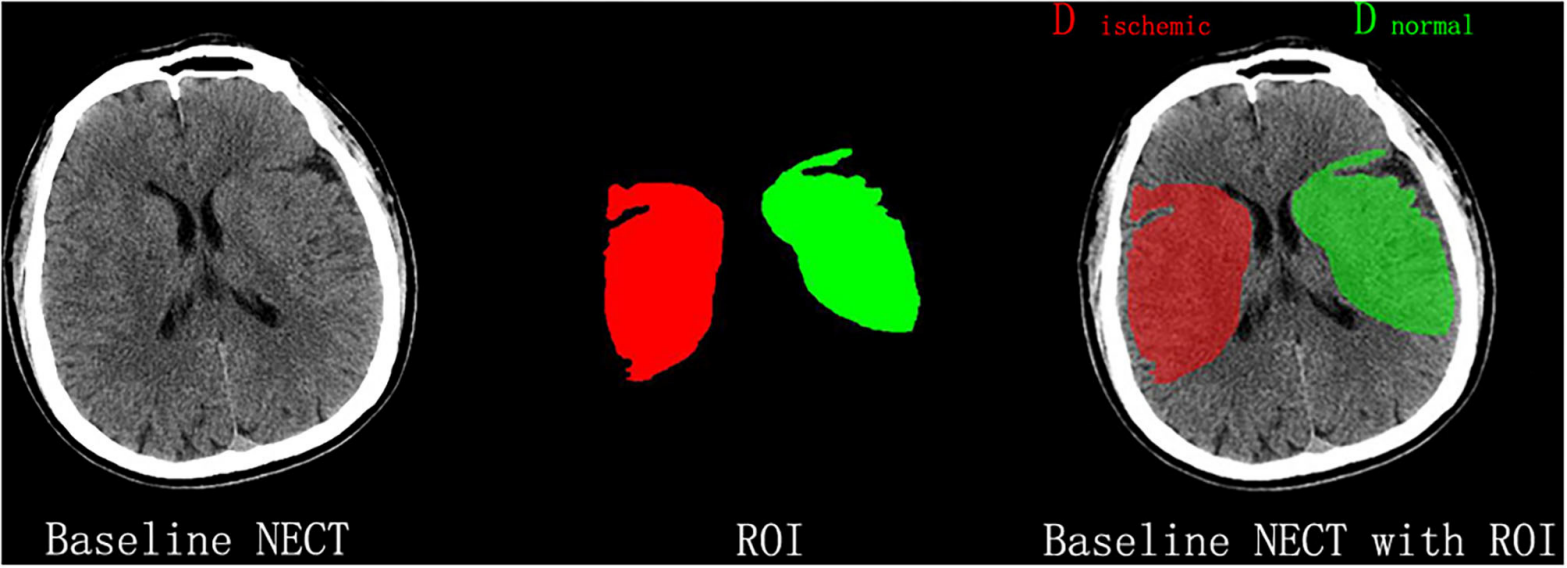
Measurement of NWU on admission nonenhanced computed tomography (NECT). According to the clinical manifestations, the infarct lesion occurred in the right middle cerebral artery territory, which was blindly outlined. The mean density of the territory (Dischemic) and a mirrored contralateral region of interest (Dnormal) was then measured to calculate NWU.

All NECT images were outlined and calculated by one experienced neuroradiologist (BQ Yang) and neurologist (HS Chen), separately. The territory of MCA at basal ganglia level was blindly outlined in a standard manner at the infarct side according to clinical evidence. As shown in Figure 1, the extension line of the anterior horn of the lateral ventricle was set as the anterior boundary, and the extension line of the posterior horn of the lateral ventricle as the posterior one, while the outer edge of the brain cortex was set as the outer boundary, and inter edge of the caput nuclei caudate and posterior limb of the internal capsule as the inter boundary. Then, the mean density of the outlined MCA territory was measured and recorded as D_ischemic_, while the mean density of a mirrored region at the contralateral side was measured and recorded as D_normal_ using commercially available software (Analyze 14.0, Biomedical Imaging Resource, Mayo Clinic, Rochester, MN). Both density measurements were sampled between 20 and 80 Hounsfield units to avoid intracranial calcifications and cerebral fluid and were eventually used to calculate NWU according to the method reported in a previous study (9).

$$\begin{array}{l} NWU\left(\%\right)\;=\;{\left(1-D_{\text{ischemic}}/D_{\text{normal}}\right)}^{*}100 \end{array}$$

### Statistical Analysis

Scatterplot was carried out using the open source statistical software RStudio Version 1.3.1093 (Rstudio PBC, Boston, MA), and visualization with the R package ggplot2 (23). Other statistical analyses were performed using SPSS 22.0 for Windows (IBM Corp, Armonk, NY). Quantitative variables were described as mean ± standard deviation (SD), or median and interquartile range (if not normally distributed) while counting data were presented as n (%). To compare the data between the two groups, we used a *t*-test or Mann–Whitney *U*-test (not normally distributed) for quantitative data, and a Chi-square test for counting data. Multivariable logistic regression analysis was used to identify the independent factors associated with ME. The odds ratio (OR) and 95% confidence interval (*CI*) were also calculated. A *P*-value of < 0.05 in two tails was considered to be significant. Receiver operating characteristic (ROC) curves and area under curve (AUC) were calculated, respectively, to assess the ability of the factors in identifying patients with ME.

The whole cohort was separated into six groups according to time windows (the time from onset to first image), that is 0–3 h, 3–6 h, 6–9 h, 9–12 h, 12–15 h, 15–24 h. ROC curves were used to assess the predictive ability of NWU in each time window in identifying ME. In addition, a scatterplot was used to show the possible relationship between NWU and the time from onset to first image. Two models reported in a previous study (9) were used to investigate if NWU is more likely to be linear-related with the time from onset to first image in 24 h. One model is NWU= b^*^ time, stating that NWU is linear-related with the time from onset to first image. The other is NWU= b^*^ log (time+1), representing a non-linear relation. Thus, NWU/time and NWU/log (time+1) were also tested together with absolute NWU as predictors of ME (9).

## Results

**Figure 2:**
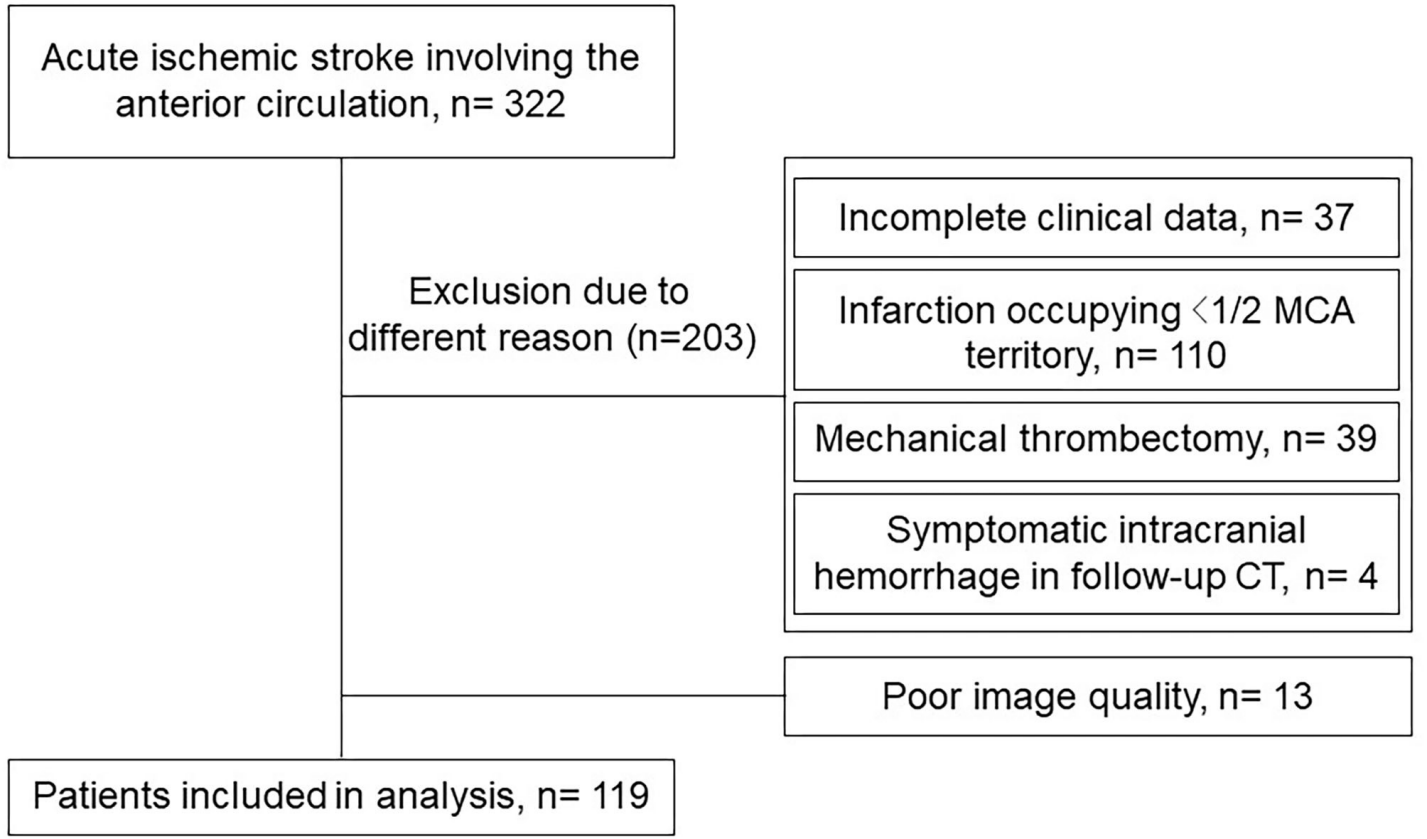
Flow chart with application of inclusion and exclusion criteria.

We continuously screened 322 patients from our stroke registry database. We excluded 203 patients due to different reasons including incomplete clinical data, infarction occupying <1/2 MCA territory, mechanical thrombectomy, symptomatic intracranial hemorrhage in follow-up CT (Figure 2). Finally, 119 patients were recruited for the analysis including the non-ME group (*n* = 87) and ME group (*n* = 32). As shown in Table 1, NIHSS at admission, the time from onset to first image, NWU in ME group were higher than those in the non-ME group (*P* < 0.05), while there is no significant difference in other characteristics between two groups.

**Table 1:** Comparison of baseline characteristics between non-ME and ME groups.

**Characteristics**	**Non-ME group (*n* = 87)**	**ME group (*n* = 32)**	***P*-value**
Female (%)	31 (35.63)	14 (43.75)	0.418
Age, mean (SD)	66.69 (±11.82)	68.56 (±10.54)	0.432
NIHSS at admission, median (IQR)	14 (11–18)	18 (13–20)	0.003
IVT (%)	35 (40.23)	9 (28.13)	0.225
Atrial fibrillation (%)	39 (44.83)	16 (50)	0.616
Hypertension (%)	48 (55.17)	21 (65.63)	0.306
Diabetes (%)	18 (20.69)	7 (21.88)	0.888
Pre-ischemic stroke (%)	15 (17.24)	3 (9.38)	0.288
SBP, median (IQR)	162 (145–177)	161 (148–185)	0.484
DBP, median (IQR)	90 (80–97)	90 (82–109)	0.332
Blood glucose, median (IQR)	7.72 (6.25–9.77)	7.56 (6.11–9.23)	0.958
OFT, median (IQR)	4.28 (2.23–8.02)	7.01 (2.65–13.55)	0.039
NWU, median (IQR)	4.65 (1.51–7.41)	9.74 (3.24–12.52)	< .0.01

*SD, standard deviation; NIHSS, National Institutes of Health Stroke Scale; IQR, interquartile range; IVT, intravenous thrombolysis; SBP, systolic blood pressure; DBP, diastolic blood pressure; OFT, time from onset to first image; NWU, Net Water Uptake*.

The multivariable logistic regression analysis showed that NWU (OR: 1.168, 95% CI: 1.041–1.310) and NIHSS (OR: 1.121, 95% CI: 1.006–1.250) were significantly associated with ME, but not the time from onset to first image and tPA thrombolysis (Table 2).

**Table 2:** Multivariable logistic regression analysis of NWU, NIHSS at admission, the time from onset to first image, and IVT.

**Factors**	**B**	**S.E**.	**OR**	**95% *CI***	***P*-value**
NWU	0.155	0.059	1.168	1.041–1.310	0.008
NIHSS at admission	0.115	0.055	1.121	1.006–1.250	0.038
OFT	0.005	0.049	1.005	0.913–1.105	0.925
IVT	−0.362	0.501	0.696	0.260–1.860	0.470

*NIHSS, National Institutes of Health Stroke Scale; NWU, Net Water Uptake; OFT, time from onset to first image; IVT, intravenous thrombolysis; OR, odds ratio; 95% CI, 95% confidence interval*.

**Figure 3:**
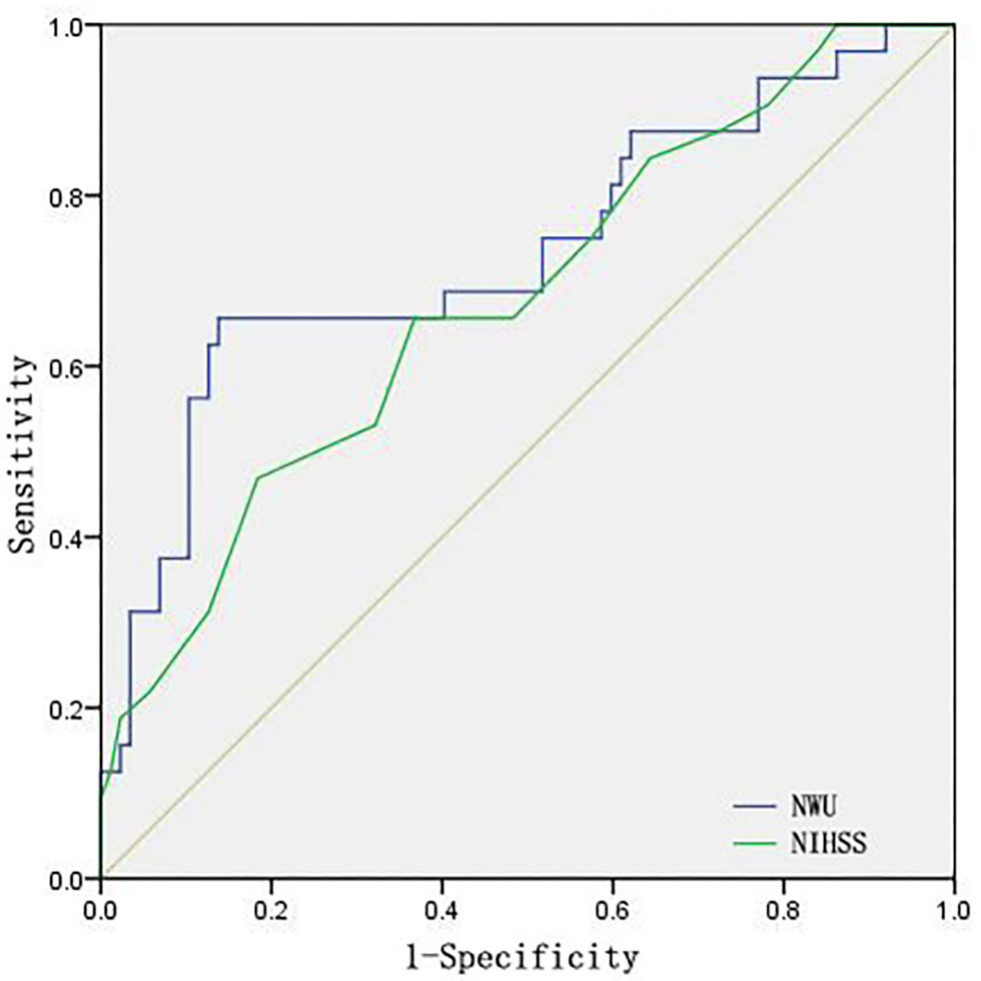
ROC curves of NWU and NIHSS at admission.

ROC curves and AUC were also calculated to assess the prediction power of the factors mentioned above in identifying patients with ME. As shown in Figure 3; Table 3, NWU showed better performance at predicting the occurrence of ME with a higher AUC (0.734) than that of NIHSS at admission. The cutoff value, sensitivity, and specificity were exhibited in Table 3.

**Table 3:** Predictive values of AUC.

**Factors**	**AUC**	**Cutoff**	**Sensitivity**	**Specificity**
NWU	0.734	8.127	0.656	0.862
NIHSS at admission	0.677	17	0.656	0.632

*NIHSS, National Institutes of Health Stroke Scale; NWU, Net Water Uptake; AUC, area under curve*.

To investigate the possible association of the time from onset to first image with the prediction ability of NWU, the patients were further divided into six groups according to time windows. As shown in Table 4, NWU in the time window of 3–6 h showed a poor predictive value (AUC: 0.500).

**Table 4:** AUC of NWU in different time windows.

**Time window**	**AUC**	***N***
0–3 h	0.649	39
3–6 h	0.500	33
6–9 h	0.788	17
9–12 h	0.958	10
12–15 h	0.778	10
15–24 h	1.000	10

*Time, time from onset to first image; AUC, area under curve*.

**Figure 4:**
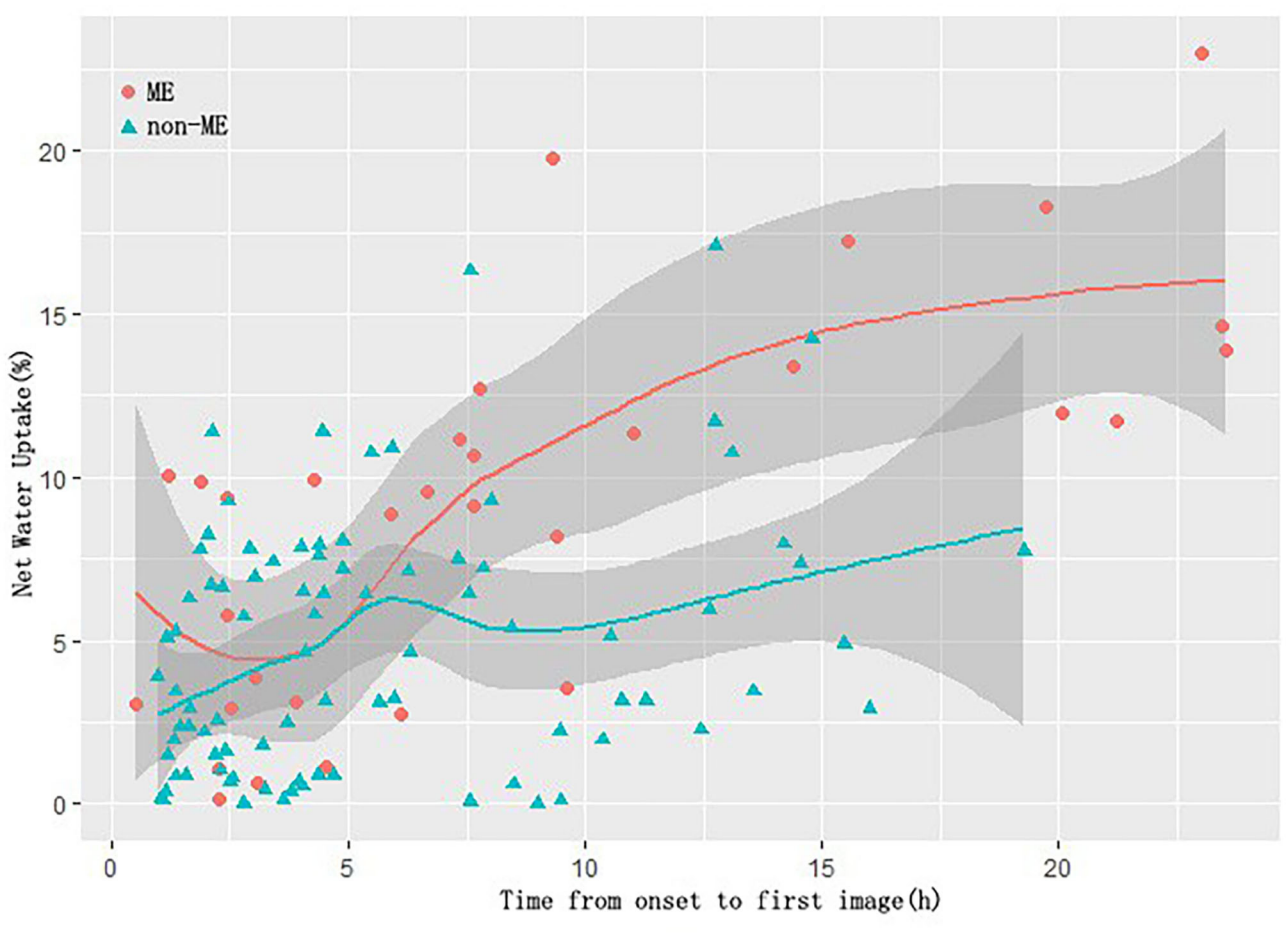
Scatterplot of NWU vs. the time from onset to first image.

Similar to the results of AUC, when the time from onset to first image was settled at the period from 3 to 6 h, the fitting line of the ME group and the non-ME group was too close to discriminate (Figure 4), suggesting that there may exist the time window of NWU predicting the occurrence of ME.

**Figure 5:**
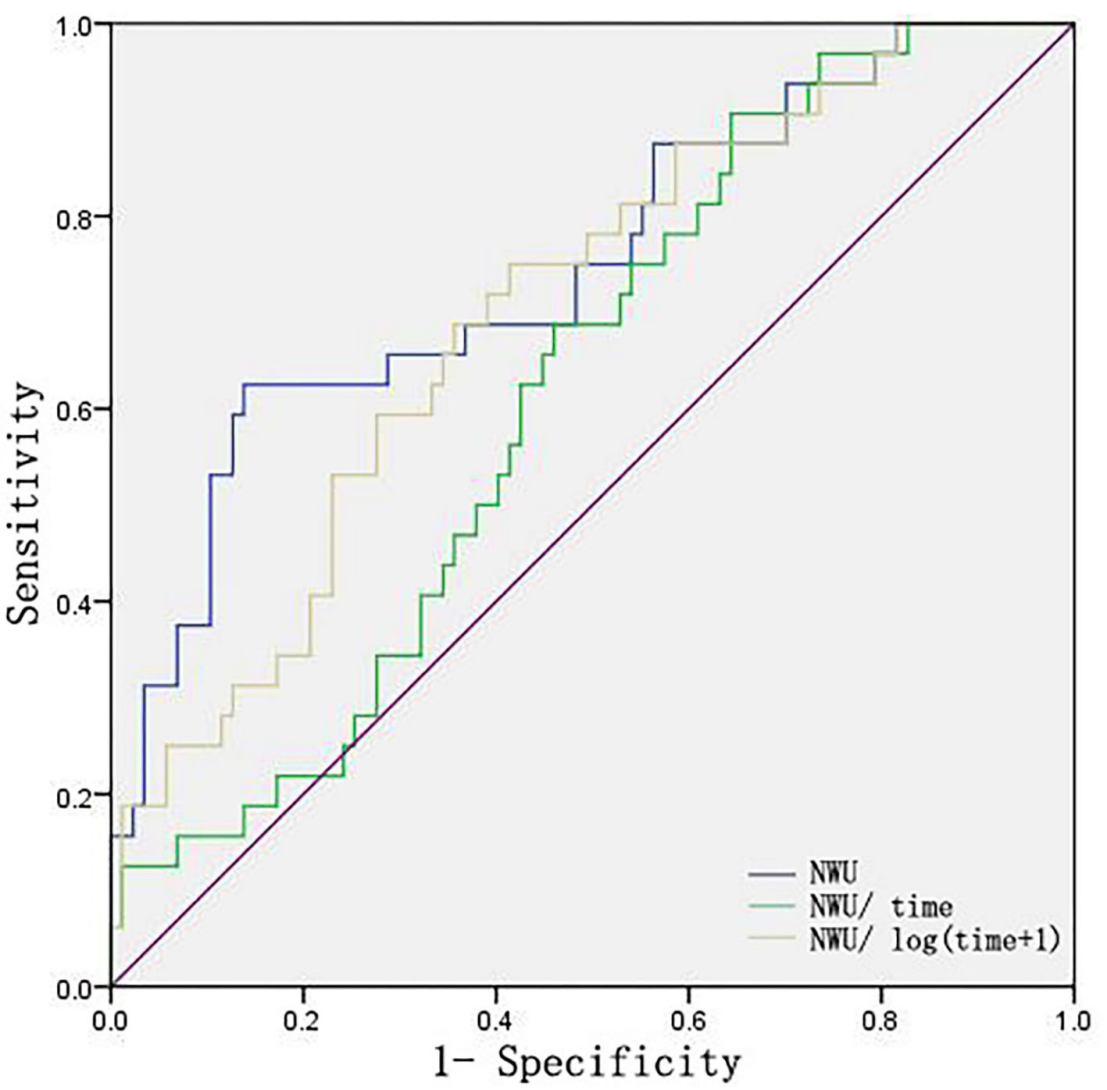
ROC curves of NWU, NWU/time, NWU/log (time+1). Time indicates the time from onset to first image.

Absolute NWU, NWU/time, and NWU/log (time+1) were also assessed through ROC curves and used to identify patients at risk of ME. As shown in Figure 5, NWU and NWU/log (time+1) exhibited better predictive power than NWU/time (NWU: 0.734, NWU/log (time+1): 0.676, NWU/time was 0.575).

## Discussion

Recent studies have shown that NWU is an important surrogate marker for the development of ME (9, 18, 19), but NWU calculation depended on CTP, which was used to detect the infarction area. The current study is the first attempt to calculate NWU based on the standardized and blindly outlined regions of MCA. The results showed that NWU calculated with this method also exhibited a good predictive value (AUC: 0.734) in identifying ME, although the predictive power was not as strong as that in a previous study (AUC: 0.93) (9).

The current method is not as accurate as the previous method (9) in defining the region of interest (ROI), because the ROI outlined in this standard manner did not represent the actual infarct lesion. For infarct lesions less than the whole MCA territory, the D_ischemic_ value measured in this standard manner should be higher than that from the actual infarct lesion. This may explain a lower NWU and cutoff value than those in the previous study (9). Although the predictive power in this study is not as strong as that in a previous study (9), the current study still provided a feasible method to identify the risk of ME at an early phase of stroke, especially in the primary stroke centers without CTP technique.

It is interesting to note that the current study suggested that there may exist a “time window” for using NWU to predict the development of ME, which has never been reported before. As shown by the AUC of NWU in different time windows (Table 4) and the scatterplot of NWU vs. the time from onset to first image (Figure 4), when time from onset to first image was within the period from 3 to 6 h, NWU did not perform well in discriminating ME. This phenomenon is very interesting and deserves to be determined in a future prospective study with a larger sample.

Considering the possible effect of the time from onset to first image on NWU, we further compared the prediction power of absolute NWU, NWU/time, and NWU/log (time+1) in the present study. The results showed that NWU and NWU/log (time+1) exhibited greater predictive power than NWU/time. This suggests that the dynamic change of NWU is not in a simple linear relationship with time within 24 h from symptom onset, which is consistent with the view of Pongpat Vorasayan's study (24). However, Broocks' team reported no significant difference among absolute NWU, NWU/time, NWU/log (time+1) in classifying ME within 6 h from symptom onset (9). The discrepancy may be due to the difference in the time from onset to first image (within 6 vs. 24 h) and in the NWU calculation methods (definite infarct lesion vs. blindly outlined MCA territory).

As we know, treatment options such as endovascular treatment and tPA thrombolysis may affect the development of edema (25, 26). To avoid the bias effect of endovascular treatment, patients with mechanical thrombectomy were excluded from this study. Given that the patients with tPA were enrolled in the present study, we further performed multivariable logistic regression analysis by adjusting tPA thrombolysis as a variable to exclude the potential effect of tPA. The results found that tPA thrombolysis did not affect the outcome in the current study.

There are limitations in this study. First, this is a single-center, retrospective study with a small sample size, which makes selection bias hard to avoid. The current method needs to be tested by a multi-center, prospective study with a larger sample size. Second, some data of paramount importance were missed, such as collateral status, infarct volume, dehydration indicators, and long-term follow-up of patients. Third, unbalanced baseline characteristics between two groups may impact the development of ME. To minimize the impact of unbalanced baseline characteristics, multivariable analysis was performed. Fourth, this standardized and blindly outlined method still requires manual operation. A software that can automatically measure NWU or even combine it with other factors related to ME could be meaningful.

## Conclusions

NWU calculated in standardized and blindly outlined regions of the MCA territory showed a good prediction power for the development of ME in patients with LHI. The easy method may provide a practical tool to distinguish patients at risk of ME in primary stroke centers with less or no CTP examination, and warrant further testing in the future.

## Data Availability Statement

The original contributions presented in the study are included in the article/supplementary material, further inquiries can be directed to the corresponding author/s.

## Ethics Statement

The studies involving human participants were reviewed and approved by the Ethics Committee of General Hospital of Northern Theater Command. Written informed consent for participation was not required for this study in accordance with the national legislation and the institutional requirements.

## Author Contributions

H-BX retrospectively enrolled patients and wrote the paper. Y-FS, NL, J-QW, and G-CC acquired data. H-BX and LT made the figures. B-QY and H-SC analyzed imaging data. H-SC designed the study and critically revised the manuscript. All authors approved the content of the manuscript.

## Conflict of Interest

The authors declare that the research was conducted in the absence of any commercial or financial relationships that could be construed as a potential conflict of interest.
